# Temporal patterns of everyday movement while working from home: a longitudinal study of step counts in Sweden during COVID-19

**DOI:** 10.1186/s12889-026-26937-w

**Published:** 2026-03-10

**Authors:** Alexandra Weilenmann, Sebastian Andreasson, Vasiliki Mylonopoulou, Mattias Rost

**Affiliations:** Department of Applied Information Technology, University of Gothenburg, Box 470, 40530 Gothenburg, Sweden

**Keywords:** Working from home, Remote work, COVID-19, Physical activity, Step count, Smartphone data, Longitudinal study

## Abstract

**Background:**

The COVID-19 pandemic accelerated a large-scale shift toward remote work, raising important public health questions about how changes in daily routines affect physical activity. While many studies have reported short-term declines in activity during the pandemic, fewer have examined long-term patterns using objective data, particularly in contexts with relatively soft public health restrictions such as Sweden.

**Methods:**

We conducted a longitudinal descriptive study using step count data from 1,529 Swedish participants collected via a custom-built smartphone app between March 2019 and March 2021. The app accessed one year of historical step data before and after Sweden’s March 2020 recommendation to work from home. Participants ranged in age from 18 to 75 years, and 55% were women. We combined demographic variation with detailed temporal analyses across months, weekdays, and hours to examine sustained and routine-specific **c**hanges in physical activity.

**Results:**

Participants took 16.2% fewer daily steps during the remote work period compared to the previous year, with the sharpest decline on weekdays (− 23.9%) and during morning hours. Weekend activity showed a modest increase (+ 2.9%), indicating partial compensation. The overall change was most dramatic in younger men (− 24.1%) and smallest in women aged 55 years and older. Temporal analyses revealed that the morning peak in step activity largely disappeared, consistent with the loss of commuting routines, while lunch and afternoon peaks remained visible. These changes persisted across several months and could not be explained by seasonal variation.

**Conclusions:**

Our results suggest that even in a country with less strict COVID-19 regulations, such as Sweden, everyday physical activity was affected. The data indicate that governmental policies advising, but not enforcing, work from home were followed. Remote work conditions may be associated with a substantial reduction in everyday physical activity, particularly during typical commuting hours. As hybrid and remote work remain prevalent in post-pandemic life, these behavioral patterns warrant attention in public health planning. The use of passively collected smartphone data provides a scalable approach to understanding the health consequences of long-term shifts in work routines.

**Supplementary Information:**

The online version contains supplementary material available at 10.1186/s12889-026-26937-w.

## Background

Regular physical activity is widely recognized as essential for health and well-being. According to the WHO, insufficient physical activity and prolonged sedentary behavior are among the leading risk factors for noncommunicable diseases worldwide. Everyday movement embedded in daily routines, such as walking to and from work, moving between meetings, or going out for lunch, contributes substantially to overall activity levels, even when it does not take the form of planned exercise. The COVID-19 pandemic prompted unprecedented changes to everyday life and work organization. In many countries, recommendations and restrictions were introduced to reduce physical contact, including working from home, social distancing, and limitations on mobility. A large body of research has documented reductions in physical activity and increases in sedentary behavior during this period (e.g. [24]).

Studies examining physical activity during the pandemic vary widely in their temporal scope and measurement approaches. Early investigations necessarily focused on short time spans, often comparing activity levels during a few weeks before and after the onset of restrictions [2, 22]. While valuable for capturing immediate behavioral responses, such comparisons are limited in their ability to assess sustained changes and are particularly vulnerable to seasonal confounding. Physical activity varies systematically by season and weather, with higher levels typically observed in summer than in winter [26]. Analyses spanning a full year before and after major societal changes are therefore better suited to disentangle seasonal variation from longer-term behavioral shifts. In addition to limitations in time span and sample composition, relatively few studies have examined *when* during the day changes in physical activity occur. Most analyses report daily or weekly totals, which can obscure shifts in routine-based movement. Hourly or diurnal analyses are particularly important for distinguishing between different forms of activity, such as commuting-related walking, lunch-time movement, and leisure activity. Identifying changes in these temporal patterns is critical for understanding how alterations in work organization, rather than general mobility restrictions alone, affect everyday movement and for informing time-specific interventions. Studies investigating physical activity during COVID-19 are predominantly based on different forms of self-reported data, often involving asking informants to assess their (own or their children’s) past physical activity through different forms of questionnaires (e.g. [7], [11], [12], [23]). Questionnaire-based assessments are affected by recall bias and social desirability bias, particularly when participants are asked to retrospectively estimate past behavior [9, 21]. A systematic review comparing self-reports with accelerometer data found that adults substantially overestimate their physical activity levels, particularly for moderate-to-vigorous activity [20]. Importantly, differences between self-reported and objectively measured physical activity do not only reflect measurement error, but also the fact that these approaches capture different dimensions of activity. Self-report measures may better reflect how individuals perceive activity intensity or distinguish between activity domains such as leisure, work, or transportation, whereas direct measures are particularly well suited to capturing incidental daily movement and lower-intensity activities that are difficult to recall or report accurately [20]. As a result, log-based measures such as step count are especially valuable for examining routine, everyday movement embedded in daily schedules, precisely the type of activity most likely to be affected by changes in work organization. Several pandemic-era studies relied on retrospective self-reports of pre-pandemic activity, raising concerns about the accuracy of such comparisons. Stockwell et al. [24] explicitly highlighted this limitation and called for more research based on objectively measured activity data. In response, a growing number of studies have used passively collected data from smartphones, wearables, and mobile applications to examine changes in physical activity. Large-scale analyses using smartphone step count data have demonstrated substantial reductions in walking during early phases of the pandemic across many countries [25]. Other app-based studies have provided insights into demographic differences and longitudinal trends but have often focused on short time windows or specific populations. For example, a UK study using an activity-promoting app drew from a user group likely to be more physically active than the general population [18], while a large U.S. study focused primarily on the first months of the pandemic [14].

Relatively few studies have explicitly linked changes in physical activity to remote work arrangements. Instead, physical activity is often examined alongside outcomes such as sleep, diet, or ergonomics when assessing the broader health impact of pandemic-related changes [5, 16]. Smaller accelerometer-based studies of office workers have provided detailed insights into daily time use during periods of working from home but often lack pre-pandemic baselines or are limited to narrow occupational groups [13]. A recent prospective cohort study of secondary school teachers reported increased sedentary behavior and reduced physical activity during lockdowns [27], and a recent meta-analysis concluded that telework is primarily associated with reductions in light physical activity, while effects on moderate-to-vigorous activity are less consistent [19].

In this study, we present longitudinal data collected through smartphones to understand changes in physical activity in Sweden during the COVID-19 pandemic compared to a pre-pandemic period. The Swedish case offers a particularly relevant case due to its different strategy to limit the spread of the virus: while other countries enforced regulations that restricted mobility, the Swedish government issued recommendations advising residents to keep social distancing and work from home when possible. This softer approach has been criticized (e.g. Lindström [17]), making it important to examine whether behavior nevertheless changed. Our question therefore was to what extent recommendations to work from home resulted in a change in step count. For this purpose, we designed and deployed an app that integrates data already collected on users’ devices, dating back to a period before the app was installed. We add to previous work by providing demographic data to examine differences in physical activity across age and gender, combined with detailed temporal analyses that capture changes over months, weekdays, weekends, and hours of the day. In contrast to previous work, we show not only that activity declined, but *when* and *how* those changes unfolded in relation to work-from-home practices. Rather than focusing solely on the pandemic as an exceptional event, we use this period to examine how large-scale societal changes are reflected in everyday movement patterns, dynamics that remain highly relevant as remote and hybrid work arrangements continue beyond the pandemic.

## Methods

This section describes the study design, data collection through a custom-built smartphone application, participant recruitment and inclusion criteria, and the analytical approach used to examine changes in step counts before and after the recommendation to work from home.

### Data collection

Step count data were collected using a custom-built smartphone application developed by the authors for this study. After receiving information about the study and providing informed consent, users selected a step data source (Apple Health on iOS; Google Fit or Garmin on Android and iOS) and authorized access to their existing step records. The app retrieved historical step count data, which were linked to basic demographic information (country of residence, gender, age, education, and optionally occupation) using unique participant identifiers. Step data were stored as time-stamped counts to support analyses by date, weekday, and hour. Additional details on the app interface and implementation are provided in the Supplementary Materials.

The app was released on the App Store and Google Play Store on October 16, 2020, and advertised through social media and a university press release, which led to coverage in several major Swedish media outlets. The app was available in Swedish and English. Although some participants used the app from other countries, the present analysis focuses on Swedish users in order to relate activity patterns to national COVID-19 recommendations. The app primarily targeted individuals working from home, but university students and adults aged 65 years and older were also invited to provide comparison data. Before sharing any step data, participants received information about the study, the data collected, and how to withdraw and delete their data, and provided informed consent. Participants reported basic demographic information, including age and gender, and could optionally report their occupation in free text.

**Fig. 1:**
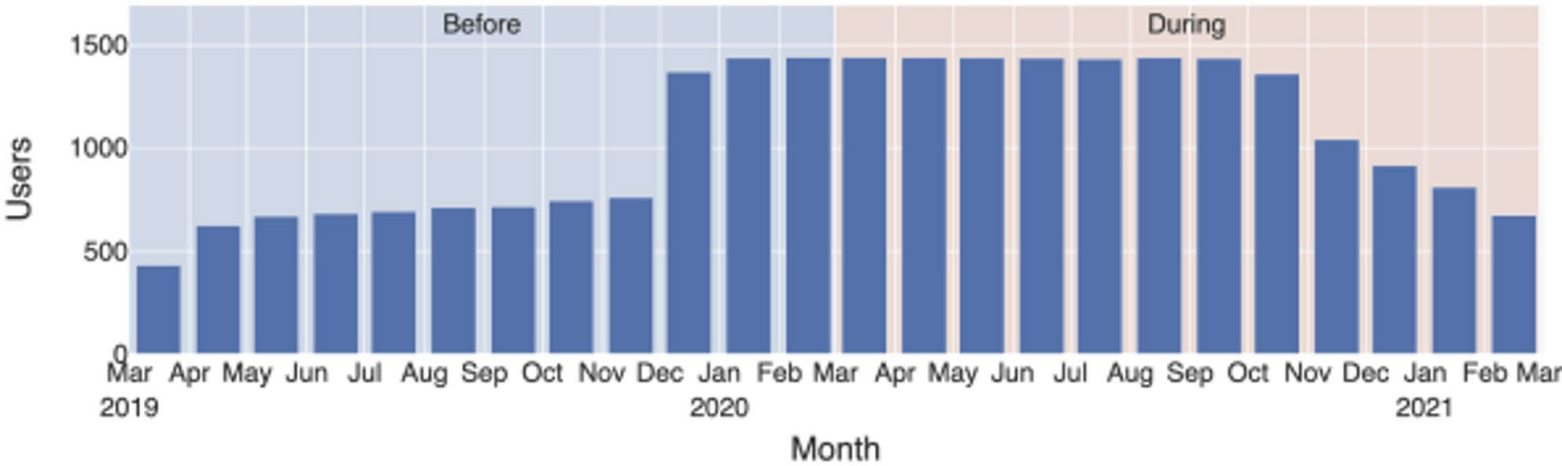
Research periods and number of users with data for each month

The app remained available for over one year, during which participants joined at different times and contributed varying amounts of historical data. For the present analyses, we included participants who had at least one complete month of step data from the year before March 16, 2020, and at least one complete month from the year after. Depending on when participants joined and their available records, this resulted in contributions ranging from one month per period to a full year. The distribution of available data across the study period is shown in Fig. 1.

**Fig. 2:**
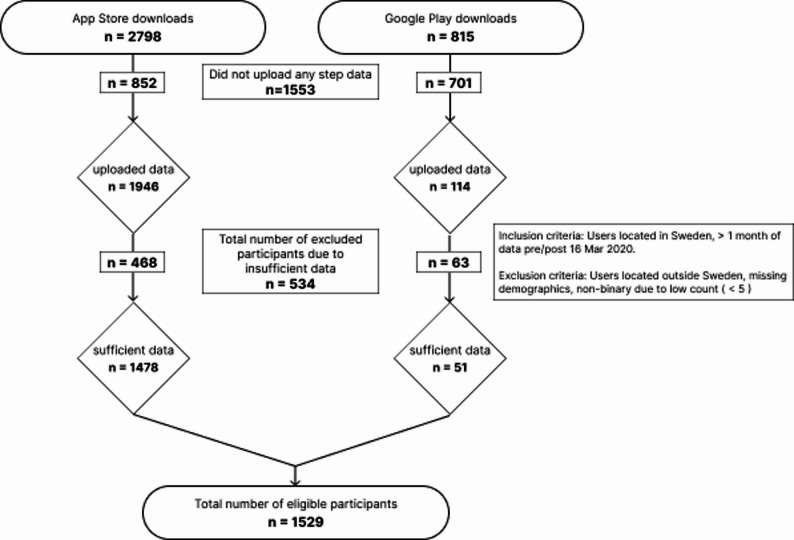
Flow diagram of participant inclusion and exclusion from app download to final analytic sample

The app got 3613 downloads (2798 iOS and 815 Android) and 2060 participants completed all the stages in the app and shared their data with us. Since we wanted to focus on the Swedish users here, we excluded 46 participants who were using the app from other countries. After a selection process, where we removed data that was incomplete or erroneous in some way, we had a sample of 1641 participants. Out of these, 112 did not fill in the initial form with their demographic data, and we were therefore left with 1529 participants (see Fig. 2 for a flow diagram of participant inclusion and exclusion). Step count data were obtained from participants’ own devices via Apple Health, Google Fit, Garmin, or Fitbit, depending on user selection. The majority of step data were contributed via Apple Health (approximately 93% of participants), with smaller proportions using Google Fit (~ 5%), Garmin (~ 1%), and Fitbit (< 1%).

### Demographic variables

Out of the 1529 participants that the analysis in this paper is based on, 848 were women (approximately 55%), 676 men and five participants selected ’other’ or preferred not to state their gender (Table 1).

**Table 1:** Descriptive characteristics of the participants

Age Group	Female	Male	Female %	Male %
18-24	77	64	54.61	45.39
25-34	86	150	36.44	63.56
35-44	160	133	54.61	45.39
45-54	230	139	62.33	37.67
55-64	162	102	61.36	38.64
65-74	60	49	55.05	44.95
75-84	17	8	68.00	32.00
85-94	0	2	0.00	100.00

### Data analysis

The app was designed to allow participants to self-report the date on which they started working from home. However, for the purposes of this study, we defined the onset of working from home using a single, policy-relevant reference date rather than individual transition dates. Many studies examining changes in physical activity during the COVID-19 pandemic use the date when the World Health Organization officially declared COVID-19 a pandemic (March 11, 2020) or other locally salient dates as comparison points. In Sweden, March 16, 2020 marks the day when national authorities formally recommended that all individuals who had the possibility to work from home should do so. We therefore used March 16 as a common cutoff date for all participants.

**Fig. 3:**
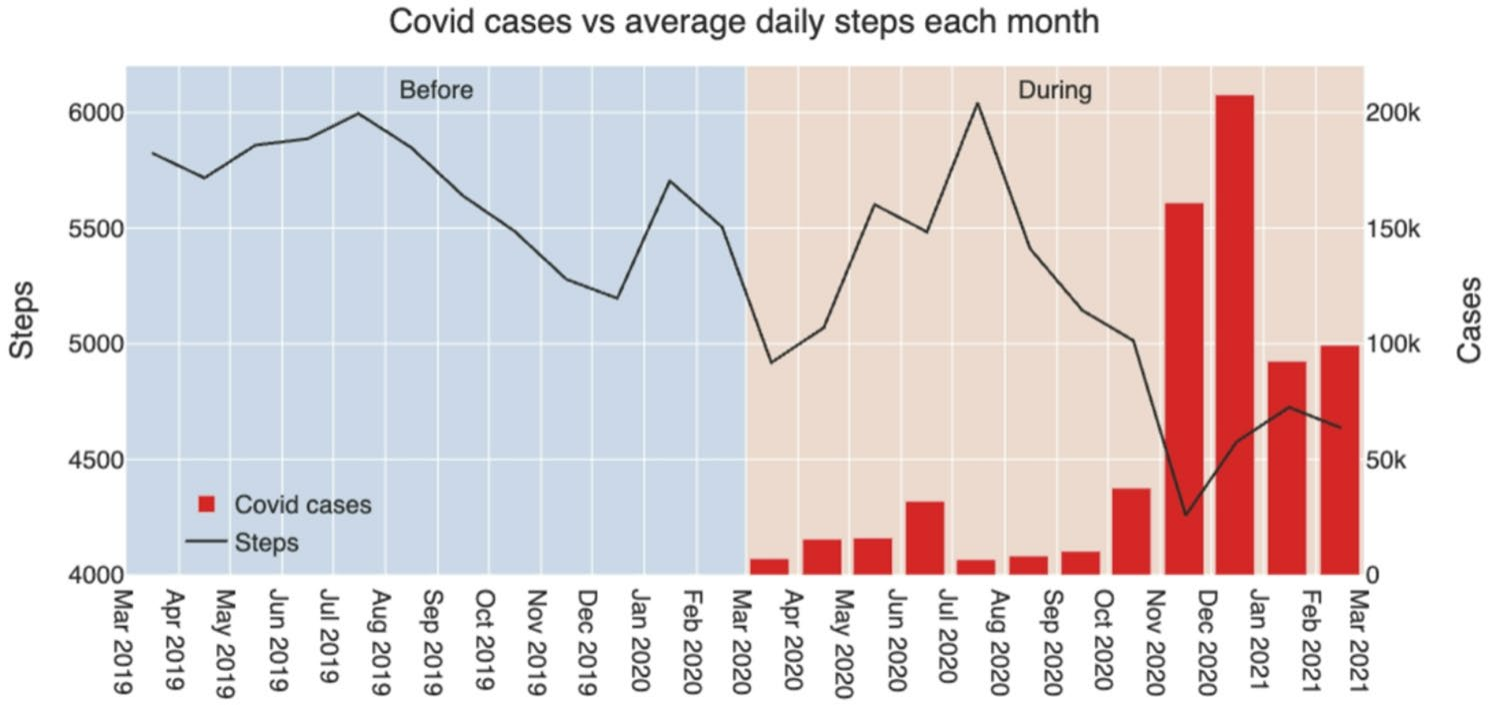
Research periods, number of reported cases of COVID-19 and step change difference

**Fig. 4:**
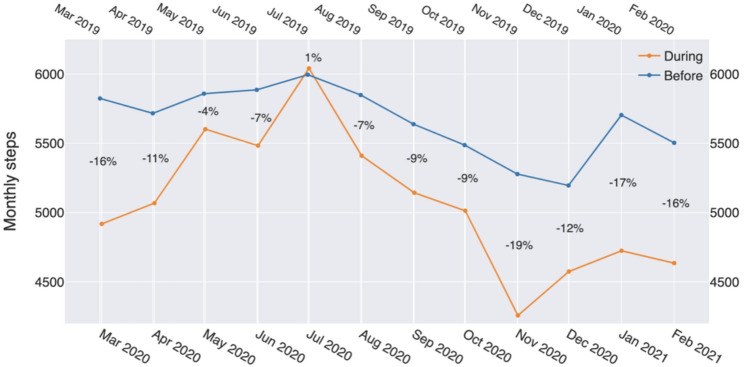
Step change differences per month for one year before and one year after Sweden’s March 16, 2020 recommendation to work from home. The percentage indicate change

**Fig. 5:**
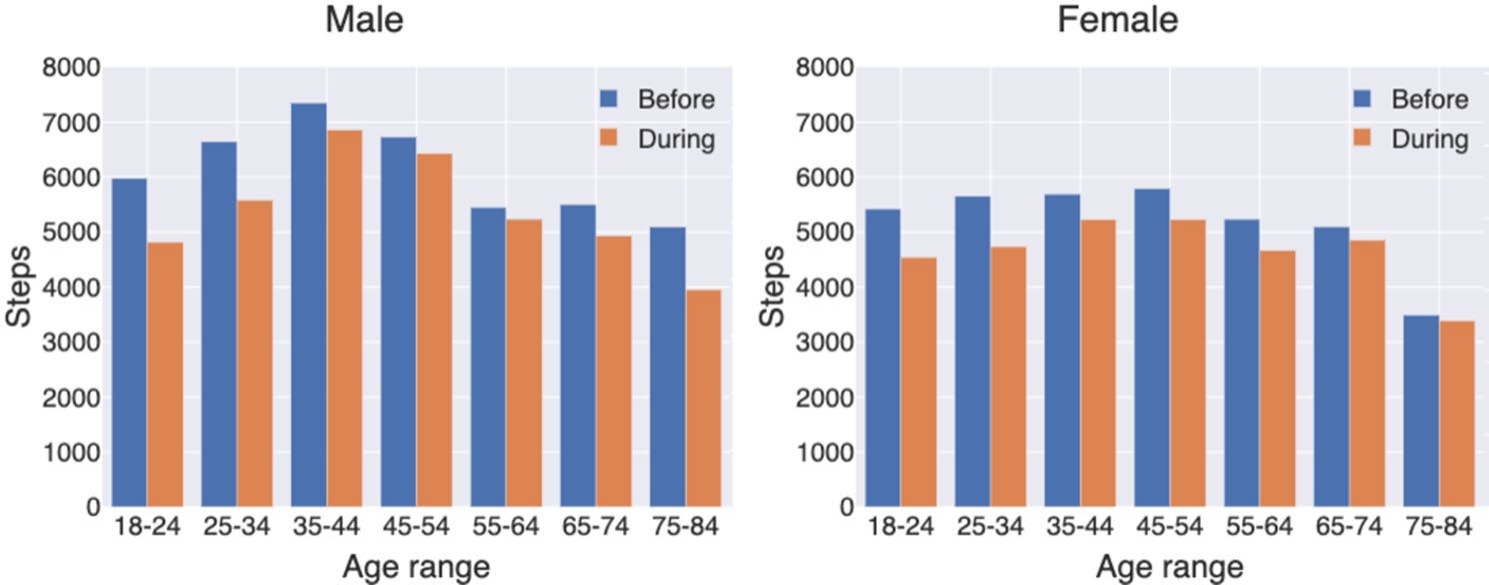
Overview of step change before and during COVID-19 for the participants

In our analysis, we examined both monthly and hourly step counts. We first constructed a dataset of hourly step counts containing participant ID, date, hour, and step count. As not all participants had data for the entire study period, we included only full months of data for each participant and excluded months with partial data. This ensured that monthly averages were based on data representing all days of the month. In our analysis, a month was defined as the period from the 16th of one month to the 15th of the next (e.g., September 2020 covers 16 September to 15 October). For each month, we calculated each participant’s average step count and then averaged these values across participants, resulting in a varying number of contributors per month (Fig. 1). Hourly (diurnal) step counts were calculated using the same principle (Figs. 3 and 4). For each participant, we calculated average steps per hour for each month, then averaged these values across participants for that month, and finally averaged hourly step counts across all months. This ensured equal weighting of each month. For Fig. 5, results are shown separately for the periods before and during the pandemic.

**Fig. 6:**
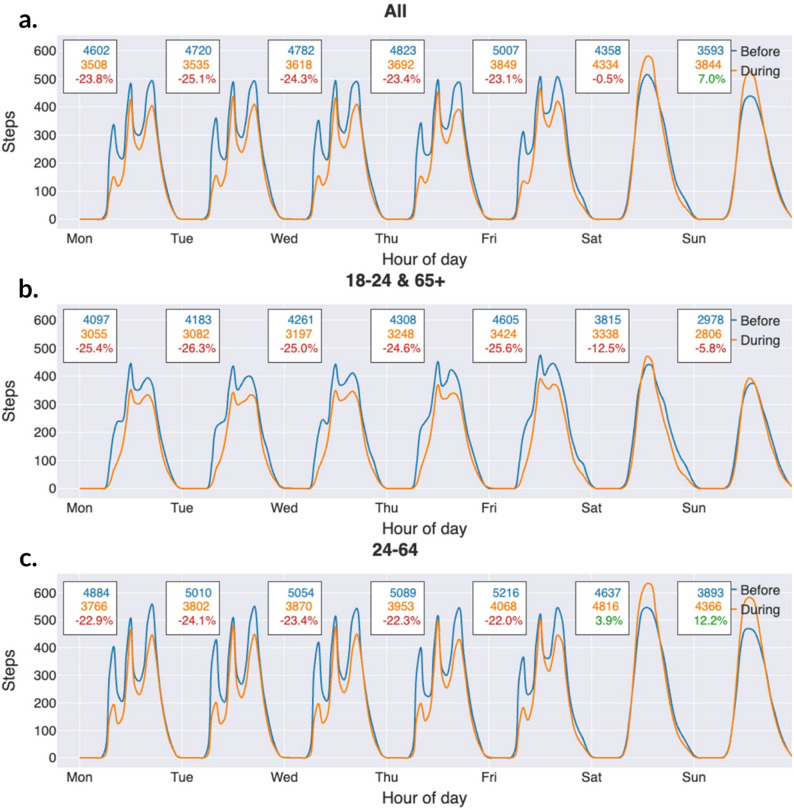
**a**, **b**, **c**: Change in number of steps for working days versus weekends. (Rendered using matplotlib, with line_shape=’spline’.)

**Fig. 7:**
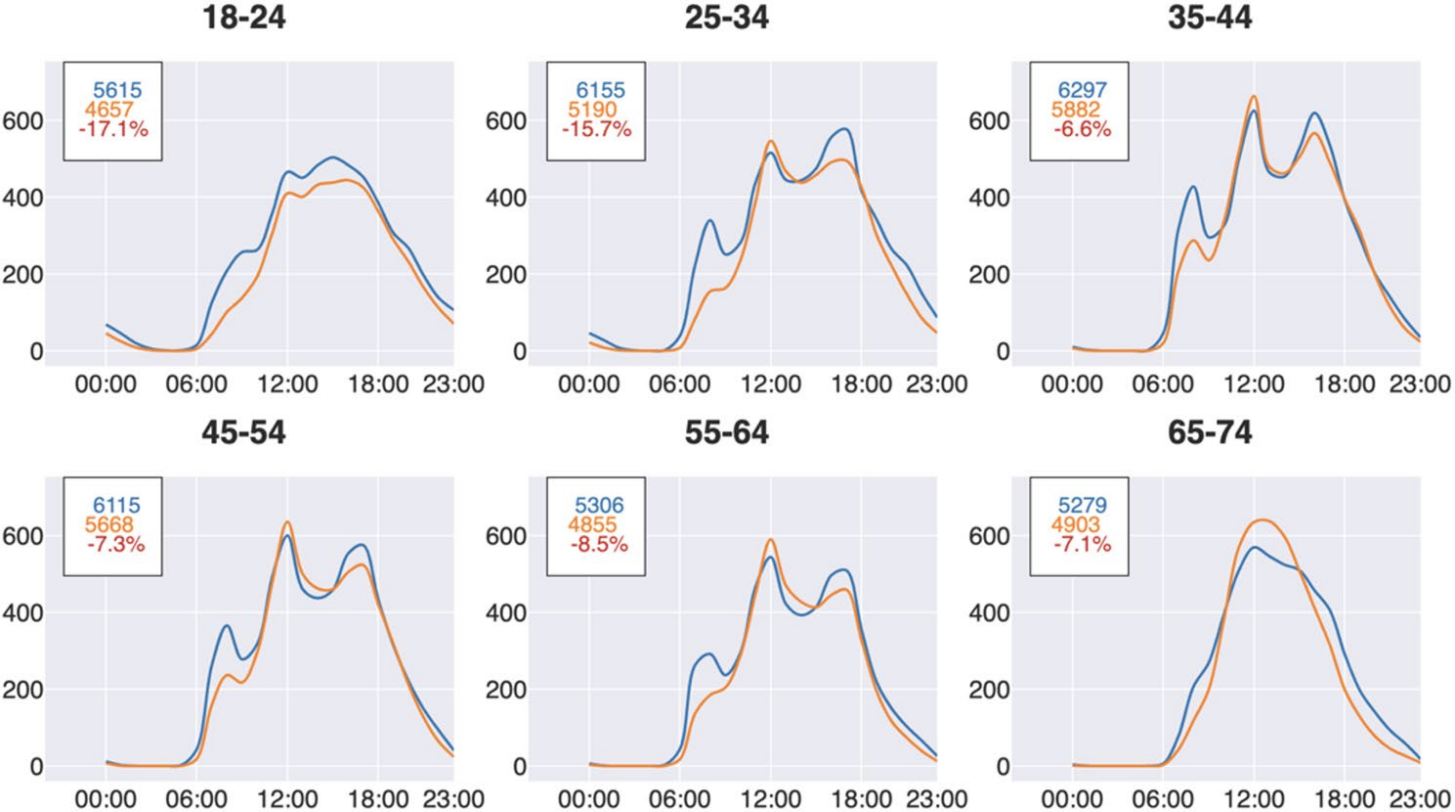
Weekdays for all ages (hour breakdown). (Rendered using matplotlib, with line_shape=’spline’.)

**Fig. 8:**
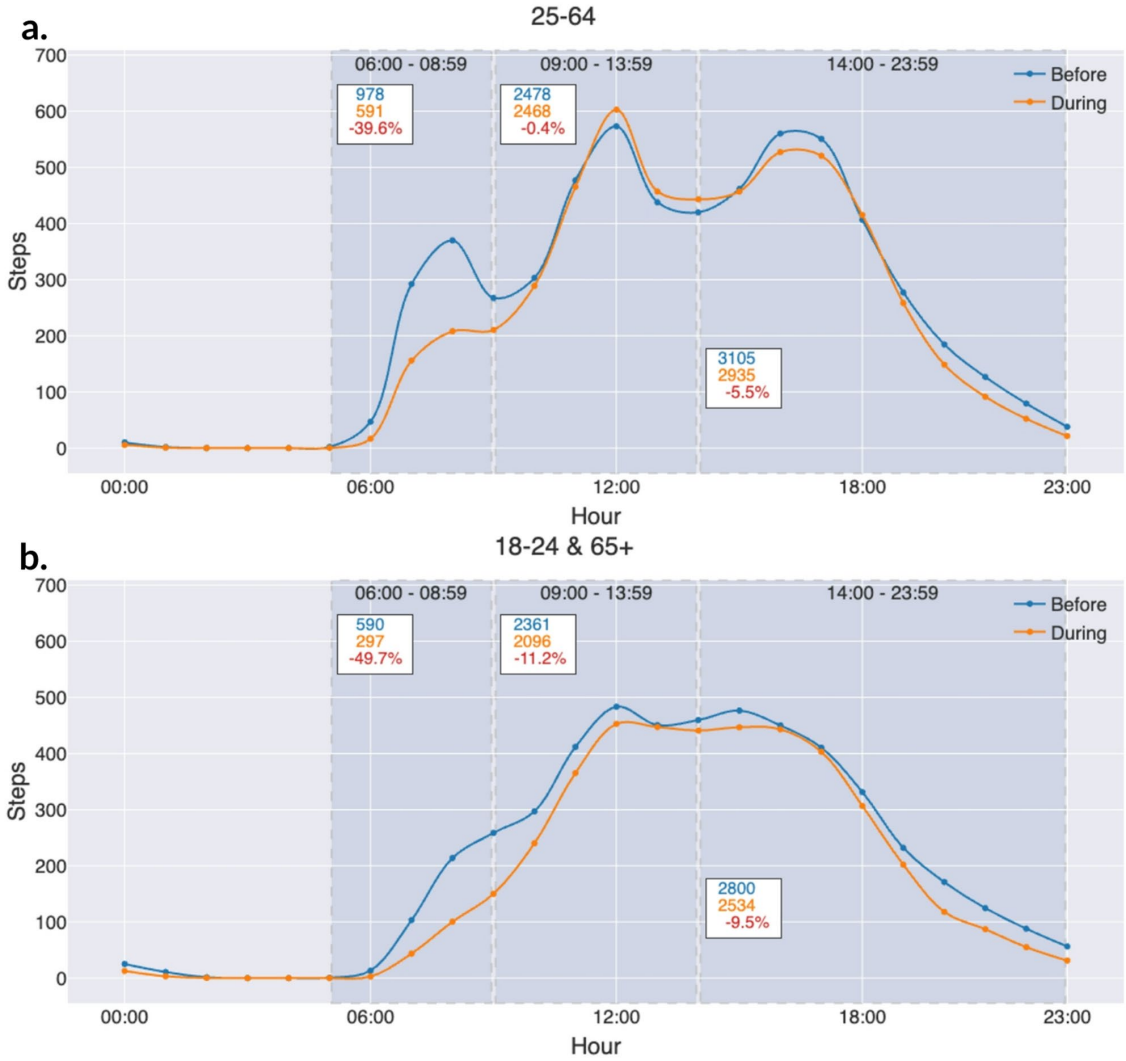
**a**, **b**: Weekdays for non-working age versus weekdays for working age participants. (Rendered using matplotlib, with line_shape=’spline’.)

The analysis was done in Python using Jupyter Notebook, NumPy, and Scipy. Smoothed line charts are rendered using spline interpolation in matplotlib (Figs. 6, 7 and 8).

## Results

In this descriptive study, we use de-identified, individual level data collected from a convenience sample through an app that we designed for this specific purpose, collecting and comparing step counts before and after Sweden’s March 16, 2020 recommendation to work from home. In the following, we report on the overall step count change for the participants, difference between weekends and weekdays, and finally the hourly step count change throughout the day.

### Overall step count decreased during the COVID-19 pandemic

The overall results show that all the participants in our study took 16.23% fewer steps during the pandemic than before. There was a general decrease across age groups and gender, but the differences varied (Fig. 5). Women had a decrease of 18.37% and men a slightly less decrease of 13.99%. The overall change was most dramatic in the group of younger men (-24.06% decrease) and the smallest change was seen in women of all age groups of 55 years and up.

From our data, it seems that the number of daily steps went down as the number of confirmed COVID-19 cases went up. Compared to the baseline year, we see a similar fluctuation over the year, with more daily steps taken during the summer than during winter (Fig. 3).

### Decrease in step count during weekdays

When it comes to daily changes in steps, our participants overall took 23.9% fewer steps during the weekdays and + 2.9% more steps during weekends (Fig. 6a). Here, we saw differences between the age groups. We made a number of simplifications, classifying all Monday-Friday as working days, and Saturday and Sunday as non-working days, based on an assumption that most people in Sweden who have jobs that allow them to work from home have this work schedule. Also, we assumed that certain age groups mostly consisted of individuals who work, and certain age groups of individuals who do not work. Those in the age span 18–24 come largely from our convenience sample of university students, so we assumed that they were not working. For the participants over 65, we also assumed that they were not working, since it is the age of retirement in Sweden. These two age groups combined had a decrease in daily step count during the week as well as weekend, but the smallest decrease was seen during Sunday (Fig. 6c).

The age group that we categorize as the working group, took fewer steps on weekdays but did in fact take more steps on weekends, particularly on Sundays + 12.2%), than before the pandemic (Fig. 6b).

### Fewer steps during morning hours

There is a clear temporal pattern during weekdays, with a morning peak in steps, a peak during lunch hour (where many Swedes go out for lunch, rather than eating in the office), and an afternoon peak (Fig. 7).

The most visible change is a decrease in the morning peak, where fewer steps are taken during the pandemic than before, both for those in the working age group and those who we assume are not working (Fig. 8a, b).

## Discussion

This longitudinal study examined changes in everyday physical activity during the COVID-19 pandemic using objective step count data from participants’ mobile devices, with a particular focus on recommendations to work from home. Conducted in Sweden, a context characterized by relatively soft, recommendation-based restrictions, our findings show that everyday movement was clearly affected, indicating that work-from-home recommendations were widely followed and had measurable public health implications. Beyond documenting an overall decline in activity, this study contributes new insight into how changes in work organization reshaped the temporal structure of everyday movement. By combining a full-year pre-post design with fine-grained analyses across hours and weekdays, we show not only that physical activity declined, but when during the day and week activity was lost, and which forms of routine movement were not recovered.

Compared to the pre-COVID-19 period, participants took on average 16.2% fewer steps, with differences across age and gender. This overall reduction is consistent with previous studies reporting decreased physical activity during the pandemic [24]. However, whereas much prior work focuses primarily on the magnitude of change, our analyses clarify the temporal organization of this decline. Compared with an earlier smartphone-based study of Swedish users reporting a 6.9% decrease in daily steps [25], we observed a larger and more sustained reduction, likely reflecting differences in sampling, demographic coverage, and observation periods. In particular, our longer baseline allows us to distinguish sustained changes in daily routines from short-term behavioral responses early in the pandemic.

Our findings align with smartphone- and device-based studies conducted in other contexts, while extending this literature through greater temporal and demographic resolution. A study of university students in Egypt and Saudi Arabia reported yearly reductions in steps during the pandemic [3], but was limited to a young, homogeneous population and coarse annual averages. In Sweden, a small accelerometer study comparing working-from-home and office days reported longer sleep but only minor differences in physical activity and lacked pre-pandemic baselines [13]. Similarly, a prospective cohort study of secondary school teachers documented increased sedentary behavior but focused on a single occupational group [27]. By combining pre-pandemic baselines, demographic breadth, and fine-grained temporal analyses, our study extends this body of work by showing not only that activity levels changed, but how these changes unfolded across the day and week over time.

One of the most pronounced patterns in our data was the reduction in weekday morning step counts, reflected in the disappearance of the pre-pandemic morning peak, most prominently among participants of working age. Few studies report hourly changes in physical activity; one notable exception is Hunter et al. [14], who similarly observed reduced morning activity. Our findings build on this work by situating these hourly shifts within a longer temporal frame and a different policy context. While lunch and afternoon peaks persisted among working-age participants, they were less evident among those assumed not to be working. A recent meta-analysis concluded that telework primarily reduces light physical activity [19]; our hourly analyses extend this conclusion by showing that much of this reduction is concentrated in morning commuting hours, a form of light, routine activity that disappears when work is relocated to the home.

In addition to diurnal changes, we observed a substantial decline in weekday step counts, which was only partly offset by increased activity on weekends, particularly on Sundays. This pattern suggests limited compensation for lost work-related movement. While U.S.-based research has shown increased recreational walking as the pandemic progressed [14], our findings indicate that routine, distributed movement embedded in workdays is not easily substituted by leisure-time activity. Importantly, these patterns persist across a full year of observation, allowing seasonal variation to be accounted for and strengthening the interpretation of these changes as structural rather than transient. While these findings are grounded in the pandemic context, they speak more broadly to how changes in work organization shape everyday movement patterns.

### Implications beyond the pandemic context

A key methodological consideration highlighted by this study is the use of app-based approaches to capture changes in everyday movement in relation to work practices. Passively collected smartphone data make it possible to examine how physical activity unfolds across days, weeks, and hours, and how these patterns shift as work routines change. Such approaches are particularly well suited for studying work arrangements that are not static, but evolve over time, including hybrid and remote forms of work. Beyond their value for research, app-based tools can also support individuals’ understanding of how changes in work organization affect their own everyday movement, for example by making losses of routine activity visible and by providing feedback that can inform healthier daily routines. In this sense, app-based methods can complement traditional self-report and cross-sectional approaches by revealing changes in everyday movement that may otherwise remain invisible.

From a public health perspective, these findings highlight the importance of considering how changes in work organization affect routine, low-intensity physical activity embedded in daily life. While much attention has focused on leisure-time exercise, our results indicate that a substantial share of everyday movement is produced through habitual activities linked to work routines, particularly commuting. Recent longitudinal evidence from multiple countries suggests that working from home has lasting implications for well-being beyond the acute phase of the pandemic [16]. As remote and hybrid work arrangements are likely to remain more common than before the pandemic [5], strategies to support physical activity may benefit from focusing on preserving or replacing lost routine movement, rather than relying solely on voluntary exercise. Targeted digital interventions and feedback mechanisms, such as those aimed at reducing sedentary time among home-office workers [6], illustrate potential avenues for mitigating more sedentary patterns as work practices continue to evolve.

### Limitations

Some limitations should be considered when interpreting the findings of this study. We treat steps as a proxy for physical activity. While step count captures everyday ambulatory movement, it does not reflect non-ambulatory activities such as cycling, swimming, or strength training, and depends on phone-carrying behavior. Validation studies indicate that smartphone-derived step counts, including those from iPhones, provide reasonably accurate estimates of ambulatory activity in free-living conditions, with modest and systematic deviations compared to criterion measures [1, 4, 15]. The findings should therefore be interpreted as reflecting changes in routine, walking-related activity rather than total physical activity.

The study relied on passively collected data from participants’ own devices, introducing heterogeneity in hardware and software. Step data were contributed primarily via Apple Health, with smaller proportions via Google Fit or Garmin, all relying on proprietary and non-transparent algorithms. As a result, absolute step counts may not be directly comparable across individuals. However, our analyses focus on within-individual changes over time rather than cross-individual comparisons, reducing sensitivity to systematic differences between platforms. Recruitment through voluntary app downloads resulted in a convenience sample that likely overrepresents health-conscious and technologically engaged individuals. Although smartphone ownership is widespread in Sweden (approximately 90% monthly active users in 2020, with more than half of the market consisting of iPhones), the sample may underrepresent groups with lower socioeconomic status or lower engagement with digital health technologies [14]. Prior research on users of health and wellness apps suggests that such samples tend to be younger, more highly educated, and of higher socioeconomic status, and to include individuals already interested in health and self-tracking [8, 10]. At the same time, this profile aligns with occupations for which remote work was more feasible during the pandemic. To reduce participant burden, we collected limited occupational and socioeconomic information and therefore could not examine variation by work role, income, working hours, or education. We classified weekdays as working days and weekends as non-working days and inferred employment status based on age groups. While this approach oversimplifies individual circumstances, observed differences in temporal activity patterns across age groups lend support to this categorization. In addition, handling of missing data introduces additional considerations. Recorded zero-step values were retained as valid observations and contributed to individual averages, whereas periods with no recorded step data were treated as unavailable and excluded from aggregation. Inclusion in the analysis required a minimum amount of complete data in both the pre- and post-periods. This approach reflects a deliberate distinction between inactivity and lack of recorded data, but may also exclude individuals with more irregular phone use and thereby influence sample composition.

Although participants were asked to self-report when they began working from home, the main analyses rely on a fixed national reference point corresponding to Sweden’s recommendation to work from home in March 2020. This enabled a common temporal anchor and visualisation of patterns across a full year before and after the onset of the pandemic, but does not capture individual variation in work arrangements, including delayed transitions, hybrid work, or continued on-site work. The results should therefore be interpreted as population-level temporal shifts relative to national recommendations rather than precise individual transition effects. In addition, app downloads and data contributions were likely facilitated by heightened public interest and engagement during the acute phase of the pandemic. Capturing activity patterns during later phases, characterized by partial returns to office work and more stable hybrid arrangements, would likely require a different study design and recruitment strategy to achieve comparable levels of participation and engagement. Finally, this study did not examine downstream health outcomes such as weight change or clinical indicators. Such data were not consistently available across platforms and would have required additional data collection beyond the scope of the study. Future research could link fine-grained, longitudinal movement data to health outcomes to further examine the longer-term health implications of changes in work routines.

Taken together, these limitations indicate that the study is best interpreted as a descriptive analysis of how everyday movement patterns shift when work routines change at scale, rather than as a definitive assessment of population-wide physical activity levels during the COVID-19 pandemic alone. While the pandemic provided a well-defined context for observing such changes, the identified temporal patterns, particularly reduced weekday and morning activity associated with the loss of routine commuting, are likely to reflect broader dynamics of how work organization influences everyday physical activity as remote and hybrid work arrangements persist beyond the pandemic.

## Conclusion

This longitudinal descriptive study shows that everyday step counts in Sweden declined during the period following national recommendations to work from home during the COVID-19 pandemic. Using passively collected smartphone data spanning one year before and one year after March 2020, we observed sustained reductions in weekday physical activity, with the most pronounced changes occurring during morning hours typically associated with commuting. Even in the absence of strict lockdowns, Swedish citizens appear to have followed governmental advice, leading to notable changes in mobility patterns. Although some increase in weekend activity was evident, this compensation was limited and did not offset the overall weekday decline.

These findings are particularly relevant as remote and hybrid work arrangements have transitioned from temporary crisis responses to more enduring features of working life. The results suggest that changes in everyday physical activity were not only short-term reactions to pandemic restrictions, but were closely tied to the loss of routine, light physical activity embedded in work-related practices, e.g. commuting. From a public health perspective, this highlights the importance of addressing *when* activity is lost, not only *how much*. As commuting-related movement decreases, strategies that intentionally support routine physical activity during workdays become increasingly important. Workplace practices, urban environments that enable short bouts of walking, and digital tools that provide feedback on changes in everyday movement all represent potential avenues for mitigating more sedentary patterns in contemporary work life.

Finally, this study demonstrates the value of smartphone-based passive data for monitoring long-term, fine-grained changes in everyday activity in periods of societal transition. While step count represents only one aspect of physical activity, log-based data can complement self-reported measures by revealing durable shifts in temporal activity patterns. Such approaches may support the design of targeted interventions and inform public health responses as work practices and daily routines continue to evolve.

## Supplementary Information

Supplementary Material 1

## Data Availability

The anonymized data set, as well as the code to reproduce our results in the figures from the aggregated data of this study, is publicly available on GitHub: (https://github.com/hci-gu/wfh-movement-jupyter/tree/2025-06-18).
