# Supplementary material for: Temporal patterns of everyday movement while working from home: a longitudinal study of step counts in Sweden during COVID-19
Source: BMC Public Health. 2026 Mar 10;26:1219. doi: 10.1186/s12889-026-26937-w (PMC13081430; doi:10.1186/s12889-026-26937-w)
Supplement: Supplementary file 1 — Supplementary Material 1 [file 12889_2026_26937_MOESM1_ESM.pdf]

Supplementary material - Temporal patterns of everyday movement while working from home: A longitudinal study of step counts in Sweden during COVID-19

App development and design

The authors of the study designed and developed a mobile application to collect and compare step count data before and after the COVID-19 pandemic in Sweden. In the following, we describe the app's core functionality relevant to data collection. Upon first launch, users completed an onboarding process explaining the study purpose, the data collected, and how the app works. Users were then asked to indicate the date they started working from home and to select a data source for step counts. On iOS, Apple Health was available; on Android, Google Fit; and on both platforms, Garmin could be selected. Users could also proceed without connecting a data source, in which case no individual-level step data were collected. When a data source was selected, users authorized access and provided informed consent before any data were uploaded to the research server. The app verified that sufficient historical data were available for comparison. While step data were uploaded, users provided basic demographic information, including country of residence, gender, age, education, and optionally occupation.

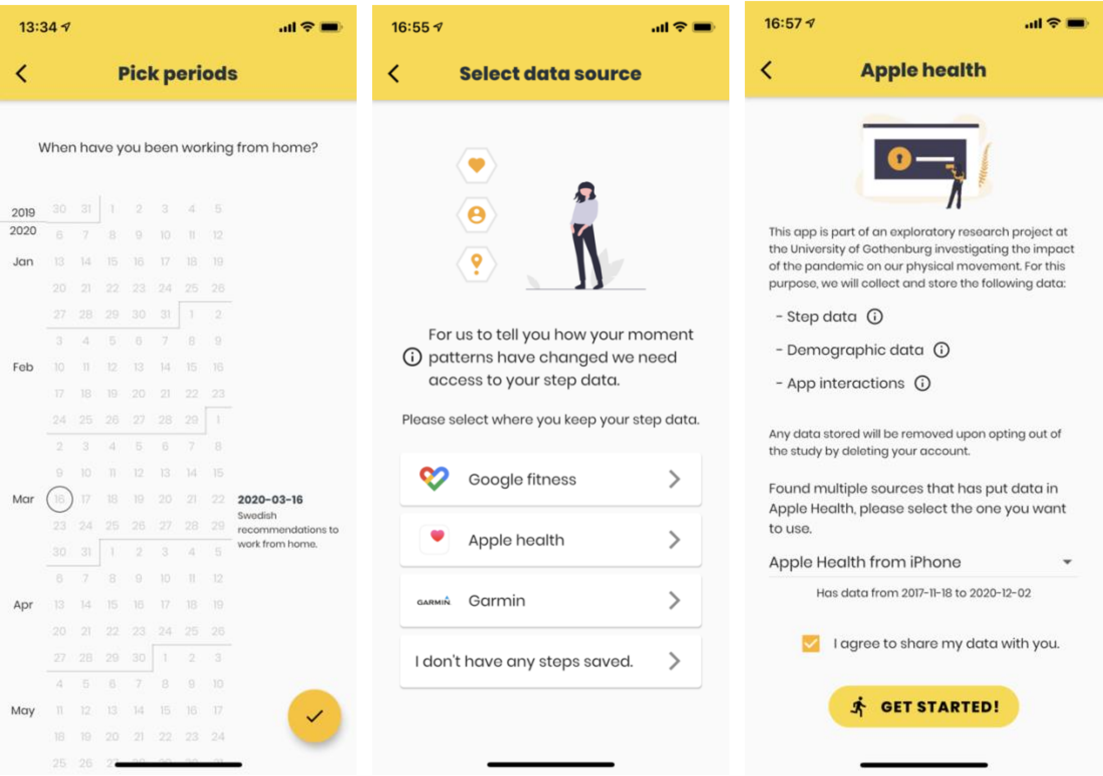

Figures 1a,b,c: The interface used to collect the step data.

The application was developed using Flutter to support both iOS and Android devices, with a backend implemented in Node.js and MongoDB. Step count data and basic user information

(demographics and self-reported work-from-home date) were stored using unique participant identifiers, allowing linkage between users and their step records. Step data were stored as time-stamped counts, including hour and weekday information to support temporal aggregation. For performance reasons, data were aggregated at the individual level to hourly step counts and to average daily step counts before and after the work-from-home period. Aggregation was performed once all uploads from a participant were complete.

Before deployment, we performed a trial where we invited a small number of users to download and use the app as a private beta through the App Store and Google Playstore, in order to evaluate functionality and the user interface.
